# Optical Properties of V_2_O_5_ Thin Films on Different Substrates and Femtosecond Laser-Induced Phase Transition Studied by Pump–Probe Method

**DOI:** 10.3390/nano12030330

**Published:** 2022-01-21

**Authors:** Yu Lan, Guowen Yang, Yangping Li, Yuheng Wang, Qianqian Shi, Guanghua Cheng

**Affiliations:** 1 State Key Laboratory of Transient Optics and Photonics, Xi’an Institute of Optics and Precision Mechanics of CAS, Xi’an 710119, China; lanyu2017@opt.cn (Y.L.); yangguowen@opt.ac.cn (G.Y.); 2 University of Chinese Academy of Sciences, Beijing 100049, China; 3 State Key Laboratory of Solidification Processing, School of Materials Science and Engineering, Northwestern Polytechnical University, Xi’an 710072, China; liyp@nwpu.edu.cn; 4 Research Center of Semiconductor Lighting and Information Engineering Technology, South China University of Technology, Guangzhou 510641, China; imtree@126.com; 5 School of Electronics and Information, School of Artificial Intelligence, Optics and Electronics (iOPEN), Northwestern Polytechnical University, Xi’an 710012, China; shiqianqian@opt.cn

**Keywords:** vanadium pentoxide, thin film, ultrafast pump–probe

## Abstract

Vanadium pentoxide can undergo a reversible phase transition by heating above 260 °C; its non-thermal phase transition, as well as ultrafast dynamical processes, is still not known. Here, femtosecond laser-induced phase transition properties in V_2_O_5_ thin films were first explored using femtosecond time-resolved pump–probe spectroscopy. The results show that the phase transient processes occur on a 10^−15^–10^−13^ temporal scale. The phase transition and recovery properties are dependent on both the substrates and pump laser energy densities. We propose the oxygen vacancies theory to explain the results, and we provide valuable insights into V_2_O_5_ films for potential applications.

## 1. Introduction

Vanadium oxides are a type of material that can undergo semiconductor–metal reversible phase transitions under the stimulation of external conditions. External stimuli include heating [1], applying a sufficient voltage or current [2], and optical irradiation [3], etc. For instance, V_2_O_5_ thin film in an initial state has high transmittance in the near-infrared band and high electrical resistivity. Under the action of some external stimuli, the transmittance or resistance of V_2_O_5_ film after phase transition drops sharply, and the process is reversible [4,5,6]. Nadkarni and Shirodkar [7] reported insulator-to-metal transition near 257 ± 5 °C in V_2_O_5_. Blum et al. [8] also reported that a V_2_O_5_ (001) single crystal surface undergoes a reversible phase transition at 77 °C–127 °C. Weiping Wang et al. [9] used a laser beam with an intensity of 255 W/cm^2^ to irradiate V_2_O_5_ film; the laser beam transmittance of the V_2_O_5_ film decreased from 51% before the phase transition to 24% after the phase transition. Of all the vanadium oxides, including VO_2_, V_2_O_3_, and V_3_O_5_, vanadium pentoxide is the most stable compound for its highest oxidation state in the V-O system, and it exhibits highly anisotropic electrical and optical properties due to its orthorhombic structure (α-V_2_O_5_) [10]. Thin-film V_2_O_5_ has attracted much attention owing to its unique electronic, chemical, and optical properties [11,12,13]. The outstanding properties of V_2_O_5_ films are of interest for use in various applications, such as electrochromic devices [14], gas sensors [15], field-effect transistors [16], supercapacitors [17], photodiodes [18], phase-change memories [19], and reversible cathode materials for Li batteries [20].

Vanadium pentoxide thin films can be prepared with different physical and chemical techniques, namely, pulsed laser deposition [21], magnetron sputtering [22], an inorganic sol–gel method [23], and spray pyrolysis [24]. Magnetron sputtering is a simpler, more controllable, and reproducible coating process compared to other methods. It has the characteristics of a fast deposition speed, small increase in substrate temperature, and small damage to the film layer. Films with a single composition, good compactness, and good uniformity can be plated by the magnetron sputtering method. The temperature-dependent phase transition properties of V_2_O_5_ thin films have been investigated by electrical and optical measurements in the past. This transition could be associated with a slight distortion of the original atomic group due to the increase in temperature, which is not accompanied by a crystallographic transformation [5]. The femtosecond laser-induced phase transition for V_2_O_5_ films has not been reported before, and the mechanism is different from the traditional thermogenic phase transition, which needs to be interpreted by our study. Time-resolved X-ray and electron diffraction spectroscopy can be used to observe the photo-induced phase transition in V_2_O_5_. Pump–probe spectroscopy is also a powerful tool to confirm the generation of metallic behavior in femtosecond and picosecond time scales after stimulated [25,26,27], which will help us to explain the ultrafast dynamical mechanism behind the phase transition of V_2_O_5_.

There are several works related to the optical and electrical properties of vanadium pentoxide thin films [28,29,30]. Few studies have been conducted on the influence of substrate materials on the photo-induced phase transition characteristics of V_2_O_5_ samples. In fact, the free surface energy and surface force are different from one substrate to another, which will affect the structure and grain distribution of V_2_O_5_ films and, ultimately, affect their phase transition characteristics. In this work, we first prepared V_2_O_5_ thin films on Al_2_O_3_, MgO, and SiO_2_ substrates by magnetron sputtering. Then, the optical characteristics of the V_2_O_5_ films at room temperature were measured by using a UV–VIS–NIR spectrophotometer and a spectroscopic ellipsometer (SE); the composition and valence of the V_2_O_5_ films were tested by X-ray photoelectron spectroscopy (XPS) and energy-dispersive spectroscopy (EDS); the surface morphology and roughness of the films were tested through an atomic force microscopy (AFM). The results show that our films have good crystal quality and high transmittance in the near-infrared band. The phase transition temperature of the V_2_O_5_ film was 260 °C, as tested by a four-point probe method. The sheet resistance measured was 990 kΩ at 197 °C and became 30 kΩ after complete phase transition. Moreover, the femtosecond laser ultrafast pump–probe method was used for the first time to directly measure the time-dependent phase transition process in V_2_O_5_. The phase transition process can occur on time scales of hundreds of femtoseconds, which was not known for V_2_O_5_ films before. Vanadium pentoxide is the highest valence oxide of vanadium and is more stable compared to vanadium dioxide. Our results can provide a possible alternative to VO_2_ films for applications such as ultrafast optical switching and laser protection for higher stability and longer reliability.

## 2. Experimental Details

### 2.1. Sample Preparation

**Figure 1:**
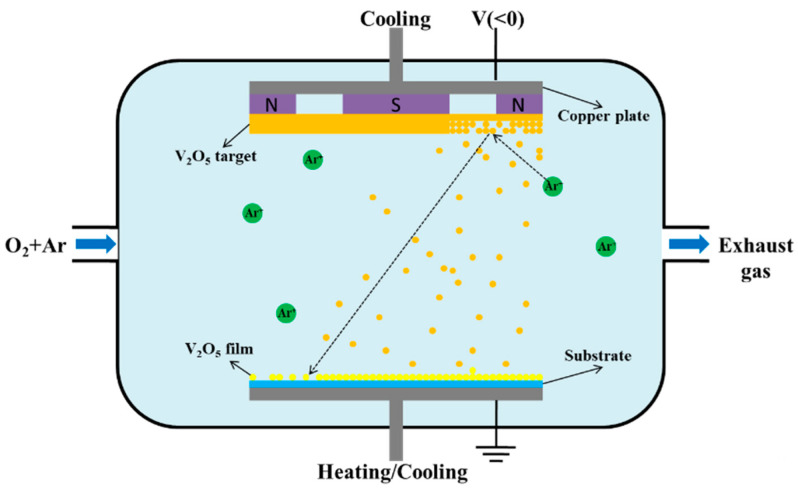
Schematic diagram of preparation of V_2_O_5_ films by magnetron sputtering.

The MSP-300BT magnetron sputtering coater was used to grow V_2_O_5_ films on amorphous (SiO_2_) and monocrystalline (Al_2_O_3_, MgO) substrates as shown in Figure 1. The thickness of these three substrates is 500 μm with double-sided polishing process. Rigorous cleaning of substrates was performed with deionized water, acetone, and absolute ethanol before coating the films, because the cleanliness of substrates has a great influence on the quality of films. The cleaned substrates were dried and placed in the sputtering chamber together with the vanadium pentoxide target (purity of 99.9%). The sputtering chamber was evacuated to 1.2 × 10^−4^ Pa, and the vanadium pentoxide target was pre-sputtered with argon gas (purity of 99.99%) to remove impurities on the surface. Oxygen (purity of 99.99%) was passed into the sputtering chamber. The flow ratio of the oxygen–argon was kept to 0.131 with the working pressure at 1.0 Pa. V_2_O_5_ thin films with a thickness of 50 nm on these three substrates can be produced by controlling sputtering times.

### 2.2. Experimental Setup

**Figure 2:**
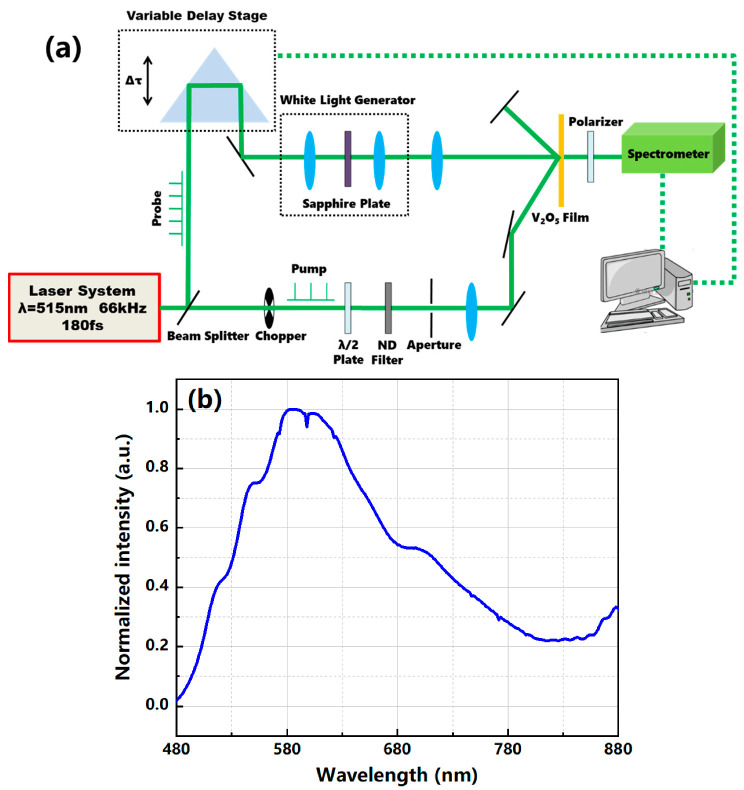
(**a**) Schematic of the ultrafast pump–probe setup. (**b**) Spectral distribution of the supercontinuum white light.

The ultrafast pump–probe setup (LIGHT CONVERSION Inc., Vilnius, Lithuania) was used for measurements of transient transmittance of V_2_O_5_ films on a 10^−13^–10^−10^ s temporal scale as shown in Figure 2a. The laser system emitted pulses of ~180 fs at a center wavelength of 515 nm and a repetition rate of 66 kHz. The laser output was split into a pump beam and a probe beam. The pump beam passed through an optical chopper with a frequency of 33 kHz and was then rotated 90° by a λ/2 plate to let the polarization direction of the pump beam become perpendicular to the polarization direction of the probe beam; this allowed for rejection of the transmitted pump beam into the spectrometer to ensure only the probe beam could be received by the spectrometer. The pump beam was then passed through a neutral-density filter to control the fluence before focusing on a ~2 mm diameter spot on the sample. The probe beam reflected off a rooftop mirror mounted on a variable delay stage to control the relative time delay between the pump and probe beams by up to 300 ps. Following the delay stage, the probe beam was focused onto a 4 mm thick sapphire plate to produce supercontinuum white light with a wavelength range of 480 nm–950 nm. The spectral distribution of the supercontinuum white light centered at ~585 nm produced by the sapphire plate is shown in Figure 2b. The probe beam was then focused onto the sample on the same spot as the pump beam but with a smaller diameter (~1 mm) using a short-focus lens to ensure probing of only the central region of the pumped region.

## 3. Results and Discussion

### 3.1. Optical Characteristics

**Figure 3:**
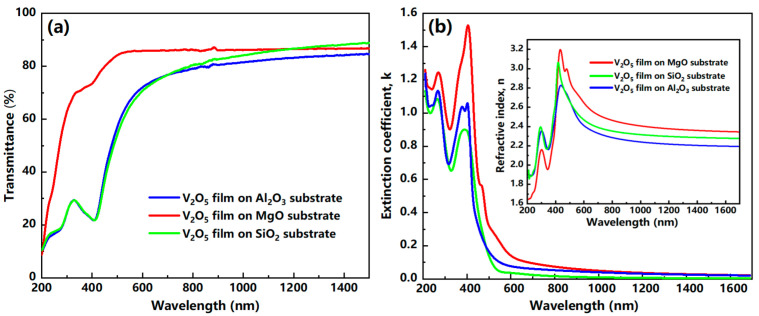
(**a**) Transmittance, (**b**) refractive index, and extinction coefficient for the 50 nm thick V_2_O_5_ films on three substrates (Al_2_O_3_/MgO/SiO_2_).

A UV–VIS–NIR spectrophotometer (SHIMADZU Inc., Kyoto, Japan) was used to measure the spectral transmittance curves of the V_2_O_5_ film samples at near ultraviolet, visible, and near infrared at 25 °C as shown in Figure 3a. A blank substrate was used to eliminate the effect of the background before conducting formal tests. The vanadium pentoxide film on the MgO substrate had a relative higher transmittance to full-band light at 25 °C than the other two substrates. Additionally, all the films of these three different substrates had a transmittance around 80% at wavelengths from 700 nm to 1500 nm at 25 °C. This shows that the V_2_O_5_ thin film we prepared has good near-infrared optical properties, suggesting that it is inseparable from the high purity and the high crystallinity of the V_2_O_5_ source. The calculated band gaps of the vanadium pentoxide films on SiO_2_, Al_2_O_3_, and MgO substrates are 2.06 eV, 2.13, eV and 2.30 eV according to their transmittance data, which are close to the previously reported results [6]. The refractive index (n) and extinction coefficient (k) were calculated by spectroscopic ellipsometry fitting. As shown in Figure 3b, the n and k for the 50 nm thick V_2_O_5_ thin films on these three substrates show the same trend with the wavelength increasing from 200 nm to 1700 nm. The parameter k is close to zero and n tends to be a fixed value (V_2_O_5_/MgO film is about 2.4, V_2_O_5_/SiO_2_ film is about 2.3, and V_2_O_5_/Al_2_O_3_ film is about 2.2) when the wavelength is greater than 1000 nm. The refractive index of the films on these three substrates reflects the structural compactness and the stoichiometry to some extent; the higher the refractive index, the closer to the characteristics of the bulk material.

### 3.2. Component and Valence

**Figure 4:**
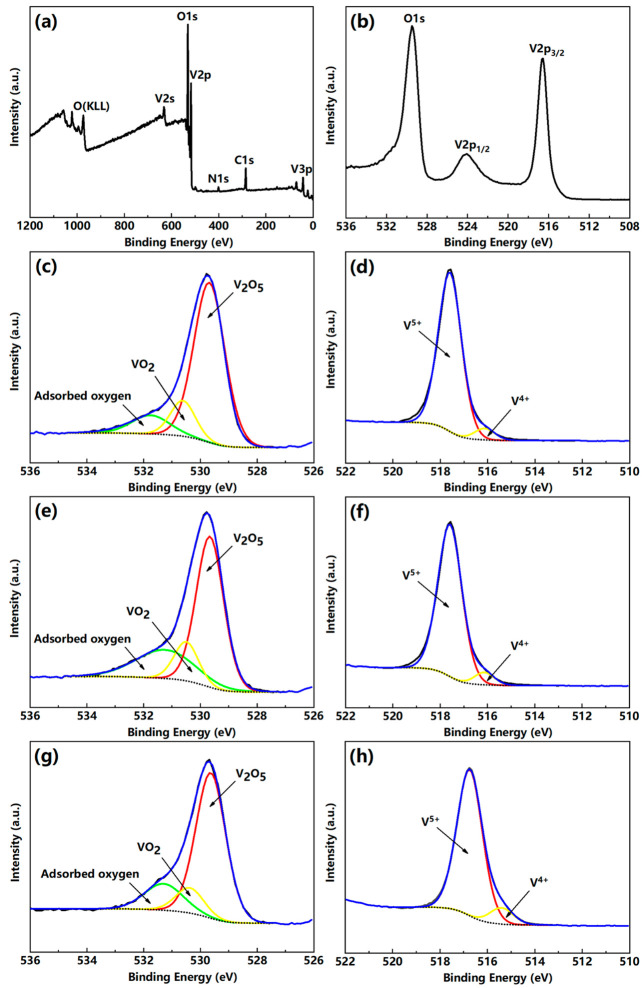
(**a**,**b**) XPS spectra and high-resolution scan of the V_2_O_5_ film on the Al_2_O_3_ substrate; (**c**,**d**) O_1s_ and V_2p3/2_ peaks fitted by Gaussian–Lorentzian curves of the V_2_O_5_/Al_2_O_3_ film; (**e**,**f**) O_1s_ and V_2p3/2_ photoelectron spectra fitting of the V_2_O_5_/MgO film; (**g**,**h**) O_1s_ and V_2p3/2_ photoelectron spectra fitting of the V_2_O_5_/SiO_2_ film.

**Figure 5:**
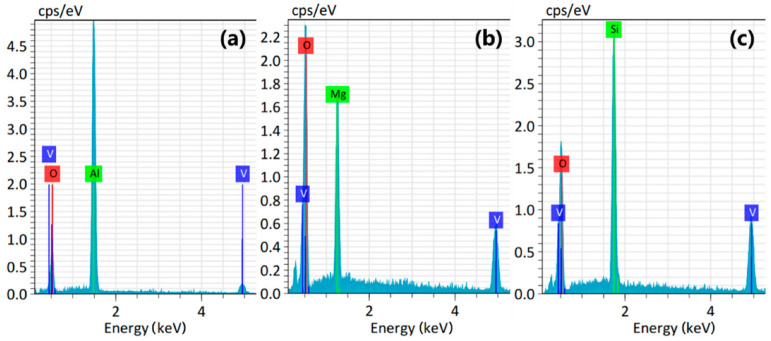
Energy-dispersive X-ray spectrometer (EDS) patterns of V_2_O_5_ films on (**a**) Al_2_O_3_ substrate; (**b**) MgO substrate; (**c**) SiO_2_ substrate.

The composition and valence state of the V_2_O_5_ film were analyzed by X-ray photoelectron spectroscopy (XPS) (KRATOS Inc., San Diego, CA, USA) as shown in Figure 4. The XPS data were obtained using an Al K_α_ monochromatic excitation source operated at 1486.6 eV, with the minimum energy resolution better than 0.45 eV. Before XPS analysis, the surface layer of the film was cleaned by Ar ion-beam etching to ensure that XPS obtained the information inside the film. According to the standard XPS database, the peak at 525.0 eV is V_2p1/2_, and the peak at 517.0 eV is the binding energy for V_2p3/2_, which is closer to the V_2_O_5_ peak V_2p3/2_. Figure 4a,b indicate that the film is mainly composed of vanadium and oxygen. Figure 4c,e,g are the O_1s_ photoelectron intensity fitted by Gaussian–Lorentzian curves for the V_2_O_5_ films on the Al_2_O_3_, MgO, and SiO_2_ substrates. They indicate that these three types of films are composed of V_2_O_5_ and VO_2_, from which we can see that VO_2_ accounted for only a small fraction of these samples. Figure 4d,f,h are the V_2p3/2_ peak curve fittings of the V_2_O_5_/Al_2_O_3_, V_2_O_5_/MgO, and V_2_O_5_/SiO_2_ samples. They mark the fitted curves of 517.5 eV and 516.1 eV corresponding to V^5+^ and V^4+^ ions, with the proportion of 95.8% and 4.2% for the aluminum trioxide-based film, the proportion of 96.1% and 3.9% for the magnesium oxide-based film, and the proportion of 94.3% and 5.7% for the silicon dioxide-based film, respectively. The XPS results show that these films are predominantly present as V_2_O_5_. Figure 5 also shows the energy-dispersive spectroscopy (EDS) analysis results of the films on these three substrates. The peaks attributed to the films and substrates can be observed in the EDS patterns.

### 3.3. Surface Topography

**Figure 6:**
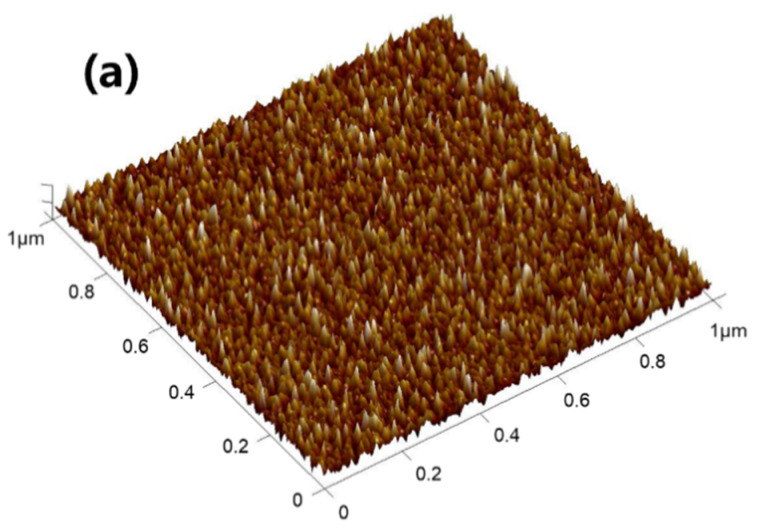

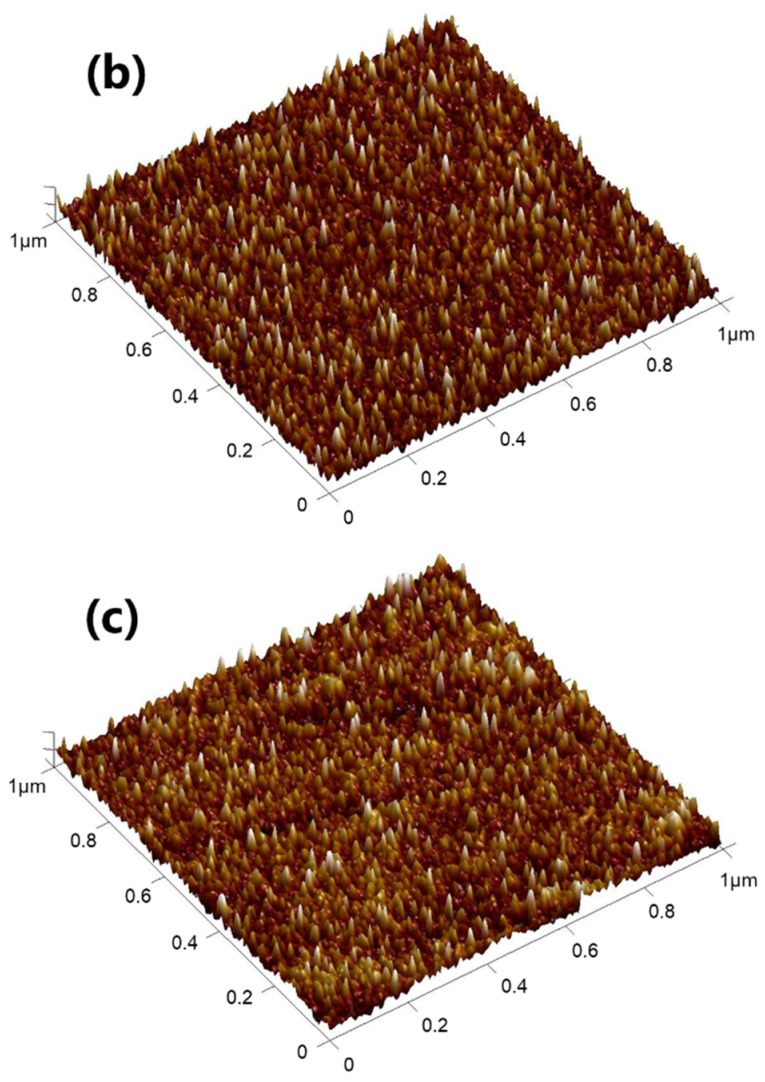
Atomic force microscopy (AFM) images (1 μm × 1 μm square scan) of (**a**) V_2_O_5_/Al_2_O_3_ film; (**b**) V_2_O_5_/MgO film; (**c**) V_2_O_5_/SiO_2_ film.

The difference in substrates directly affects the surface quality of V_2_O_5_ films. The surface quality of films is critical for their phase transition properties under laser radiation. The images obtained by atomic force microscopy (AFM) (Bruker Daltonics Inc., Billerica, MA, USA) show that the V_2_O_5_ films deposited on Al_2_O_3_, MgO, and SiO_2_ substrates have different surface topographies as shown in Figure 6. The values for the root-mean-square (rms) roughness of V_2_O_5_/Al_2_O_3_, V_2_O_5_/MgO, and V_2_O_5_/SiO_2_ are δ_V2O5/Al2O3_ = 0.551 nm, δ_V2O5/MgO_ = 0.633 nm, and δ_V2O5/SiO2_ = 0.777 nm, respectively. The growth of V_2_O_5_ thin films on different substrates must go through the following four stages [31]: (a) The atoms sputtered from the target are incident and adsorbed on the surface of the substrate. (b) The adsorbed gas-phase atoms diffuse and combine to form atom pairs or small atom groups. (c) The atom pairs or small atom groups capture other adsorbed atoms to form stable nuclei. (d) Stable nuclei continue to grow and gradually form small islands. The obvious distinctions in morphology and roughness of the V_2_O_5_ films grown through these four stages should be attributed to the differences in the free surface energy and surface force of Al_2_O_3_/MgO/SiO_2_ substrates, resulting in differences in the formation and growth of crystal nuclei.

### 3.4. Electrical Performance

**Figure 7:**
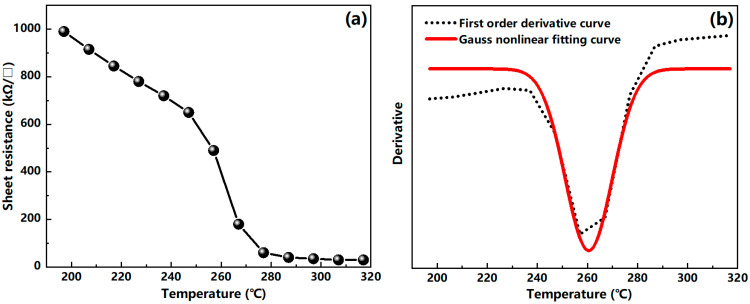
(**a**) Temperature–sheet resistance characteristic curve; (**b**) the first-order derivation (black dotted line) and the Gaussian nonlinear fitting (red line) on the temperature–sheet resistance characteristic curve.

A four-point probe method was used to test the sheet resistance of the V_2_O_5_ film. During the test, the V_2_O_5_ film was heated by a thermocouple to measure the corresponding relationship between the sheet resistance and the temperature. The phase transition temperature of vanadium pentoxide film is about 257 °C theoretically [32], so we chose the temperature range of 197–317 °C for this study. Figure 7a shows the sheet resistance of the vanadium pentoxide film with the temperature. It can be seen from the figure that the vanadium pentoxide film has a high sheet resistance value at a temperature of 197 °C, and it gradually decreases as the temperature increases, which exhibits a metal characteristic. In order to obtain the phase transition temperature of the sample, we performed a first-order derivation (black dotted line) and Gaussian nonlinear fitting (red line) on the temperature–sheet resistance curve to obtain Figure 7b. According to Figure 7b, the phase transition temperature of the sample is about 260 °C.

### 3.5. Ultrafast Nonlinear Optical Response Properties

**Figure 8:**
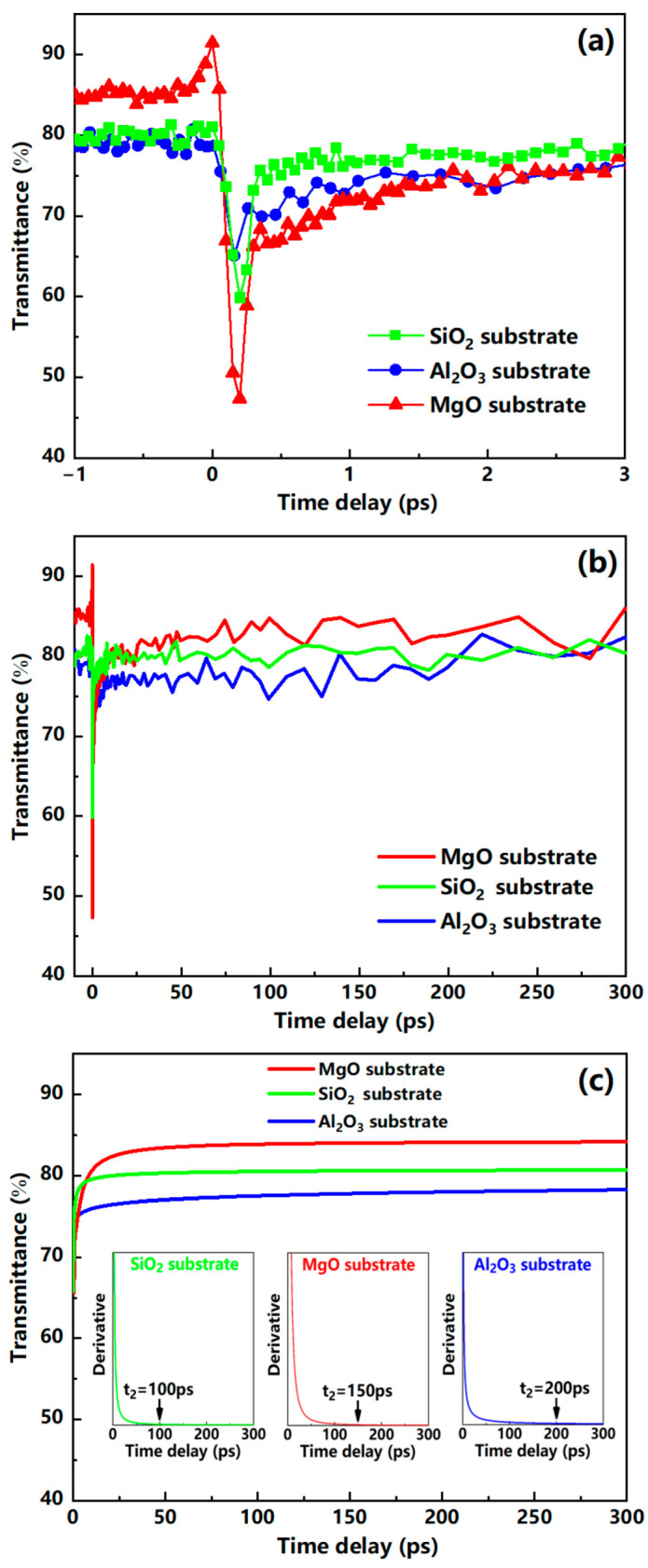
Transient transmittance changes in V_2_O_5_ films on three substrates (Al_2_O_3_/MgO/SiO_2_) induced by laser pulses with an energy density of 0.0622 mJ/cm^2^. (**a**) Partial data showing the initial response and recovery of V_2_O_5_ films; (**b**) full-track data. (**c**) Fitting and deriving (three insets) to the recovery process data to obtain the recovery time t_2_.

Transient transmittance changes in V_2_O_5_ thin films on three different substrates (Al_2_O_3_/MgO/SiO_2_); at various delay times, Δt was measured by the femtosecond laser ultrafast pump–probe method, and the results are shown in Figure 8. The pump light energy density of 0.0622 mJ/cm^2^ on the samples was sufficient to excite complete phase transition. In general, vanadium oxide thin film has a higher transmittance in the semiconductor phase and a lower transmittance in the metal phase [25,33,34]. We speculate that the rapid decrease in the transmittance of the V_2_O_5_ films is due to its transformation from the semiconductor phase to the metal phase when irradiated with the femtosecond laser. The time required for the transmittance to fall from the initial value (t = 0) to the minimum value is defined as the phase transition time t_1_. Then, the samples begin to slowly return from the metal phase characteristics to the semiconductor phase characteristics, and the transmittance begins to increase gradually. The time required for the transmittance to change from the minimum value to a stable value is defined as the recovery time t_2_. The optical response characteristics of the V_2_O_5_ thin films on these three substrates are shown in Table 1. It can be seen from Table 1 that the phase transition time of the film on the Al_2_O_3_ substrate was as short as 160 fs compared with the MgO substrate and the SiO_2_ substrate. This could be due to the AFM results showing that the V_2_O_5_/Al_2_O_3_ film has a smaller surface roughness (0.551 nm), indicating that the V_2_O_5_/Al_2_O_3_ film is more continuous and has a better surface quality, which then affects its phase transition characteristics. The observed phase transition process was too fast to be attributed to lattice temperature effects, because thermal effects cannot occur in such a short time.

In order to obtain the recovery time for these three types of V_2_O_5_ films, a nonlinear fit was performed based on the data of the recovery process. Figure 8c shows the corresponding three fitted curves. These curves all obey a power function model described as

(1) $$T=At^{B}+C$$

where *T* represents the transmittance, and *t* is the different time delays; the fitted values of *A*, *B*, and *C* for different samples are shown in Table 2. By further deriving these three fitted curves, we obtain a starting point where the derivative value is equal to zero (d*T*/d*t* = 0), which means that *T* no longer changes with an increase in the time delay, and the recovery process has been completed. The recovery time of the film on the SiO_2_ substrate after phase transition was shorter than that of the other two substrates, and it exhibited excellent recovery characteristics. From the change in the transmittance before and after the phase transition, the film on the MgO substrate was changed by 38% higher than that of the other two substrates, which indicates that V_2_O_5_/MgO film has better phase transition characteristics. The different phase transition characteristics of the V_2_O_5_ films on Al_2_O_3_/MgO/SiO_2_ substrates could be caused by the interface effect of crystals, such as the difference between ionic valence states and electron motion transmission at the interface between the V_2_O_5_ crystal and the Al_2_O_3_/MgO/SiO_2_ crystal. The reason why the transmittance T_3_ of the vanadium pentoxide films after the phase transition recovery process is less than the initial transmittance T_1_ is that some of the V_2_O_5_ grains will remain with metallic phase characteristics after complete relaxation [35].

The femtosecond laser-induced V_2_O_5_ phase transition mechanism can be explained as follows: the chemical bonds of V–O in the vanadium pentoxide will quickly break to generate oxygen vacancies and oxygen atoms when a femtosecond laser exceeding the phase transition threshold energy density is irradiated to the V_2_O_5_ film. This process can be written as

(2) $$V_{2}O_{5}=V_{2}O_{5-x}+xV_{O}+xO$$

where V_O_ represents the oxygen vacancies formed in the V_2_O_5_ crystal. The oxygen vacancies V_O_ in the crystal are ionized, forming ionization vacancies ${\dot{V}}_{O}$ and ${\ddot{V}}_{O}$ and excess electrons e′; this process can be expressed as

(3) $$V_{O}={\dot{V}}_{O}+e^{\prime }$$

(4) $${\dot{V}}_{O}={\ddot{V}}_{O}+e^{\prime }$$

Therefore, Equation (2) can be written as

(5) $$V_{2}O_{5}=V_{2}O_{5-x}+x{\ddot{V}}_{O}+xO+2xe^{\prime }$$

These excess electrons are bound by the positive center formed by the oxygen vacancy points and are in a weakly bound state, thus forming an additional donor level under the conduction band, which is closer to the bottom of the conduction band. Some of the electrons in the donor level are excited into the conduction band to become carriers. Therefore, vanadium pentoxide changes from an insulator characteristic to a metallic characteristic under femtosecond laser irradiation. When the electrons transition to the conduction band, positively charged holes will be formed at the corresponding positions of the donor level. They will recombine through radiative and non-radiative recombination on a timescale of tens to hundreds of picoseconds, and then the V_2_O_5_ phase transition will be complete. Some of the electrons are in a relatively long-lived state in the conduction band, allowing the V_2_O_5_ film to maintain the metal properties for a long time after phase transition.

**Figure 9:**
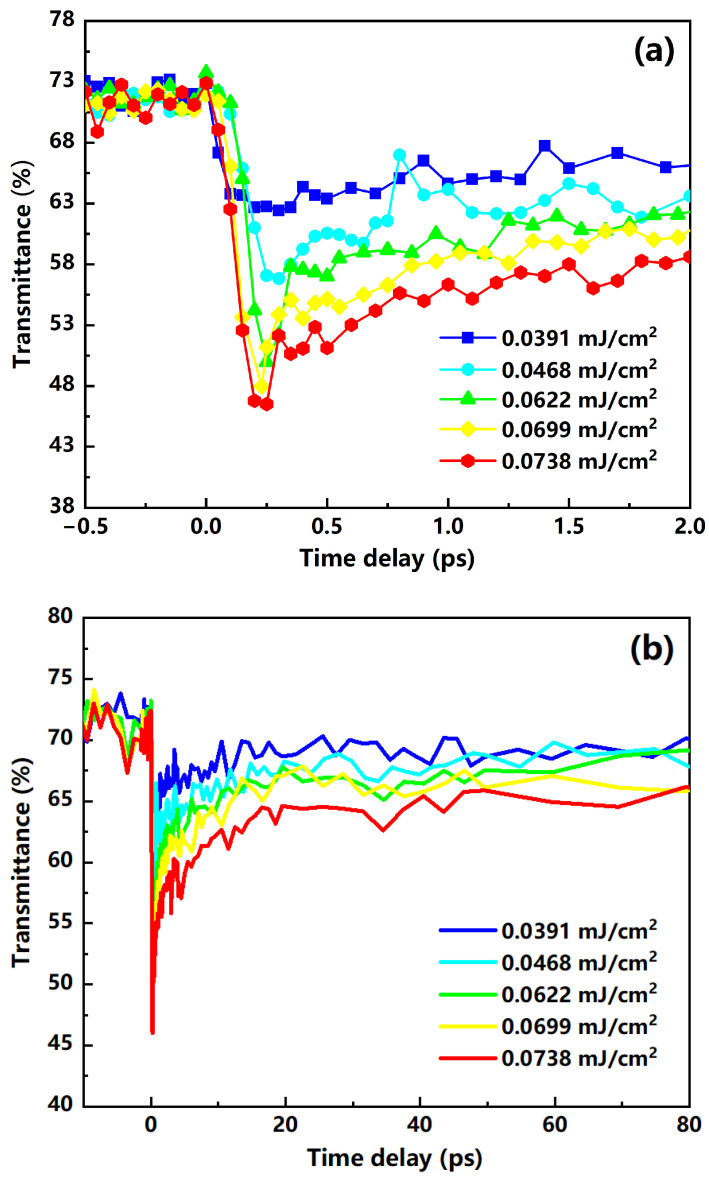
Different energy density laser pulse-induced transient transmittance changes in the V_2_O_5_ film on the Al_2_O_3_ substrate. (**a**) Partial data; (**b**) full-track data.

For the V_2_O_5_/Al_2_O_3_ film, the change in transmittance at different pump energy densities was measured by varying the time delays between the pump and probe pulses as shown in Figure 9. The pump energy density of 0.0391 mJ/cm^2^ was sufficient to cause the phase transition of the sample, and the pump energy density of 0.0738 mJ/cm^2^ was below the damage threshold for the film. In Figure 9, all the curves begin to drop sharply within 300 fs after the arrival of the pump pulse and then slowly rise within a few picoseconds. The change in transmittance from the maximum value to the minimum value indicates that the sample has fully completed the phase transition, and then the slow rise in transmittance shows the recovery process of the sample. Obviously, the sample with a higher pump energy density has a faster initial transient response, or “turn on” of the film’s response. For instance, the V_2_O_5_/Al_2_O_3_ sample has a phase transition time of approximately 250 fs when the pump energy density is 0.0738 mJ/cm^2^, which is shorter than the phase transition time at other pump energy densities (0.0699 mJ/cm^2^, 0.0622 mJ/cm^2^, 0.0468 mJ/cm^2^, and 0.0391 mJ/cm^2^). This is because the phase transition depth of the V_2_O_5_ thin film is smaller when the pump light with a lower energy density is used for irradiation. Based on this situation, only the surface portion of the V_2_O_5_ film will undergo a phase transition, and this process requires very little time. The phase transition depth of the V_2_O_5_ film becomes larger as the laser energy density continues to increase, and, thus, the V_2_O_5_ film needs more time to complete the transition. Additionally, the initial fast transient response of the V_2_O_5_/Al_2_O_3_ film becomes larger when the pump energy density is greater, which indicates that an increasing amount of V_2_O_5_ is changed to the metal phase characteristic with an increase in excitation intensity. This could be due to the fact that vanadium pentoxide produces more electrons after laser irradiation when the energy density of the pump light is higher. Excess electrons are excited into the conduction band to form more carriers, resulting in a large degree of phase transition and corresponding to a large change in transmittance. Furthermore, when the film was irradiated with a higher energy density pump light, the recovered film after phase transition had a greater ΔT (ΔT is equal to the initial transmittance at 25 °C without laser irradiation minus the transmittance after the full recovery process). The reason is that more V_2_O_5_ grains will remain with the metallic phase characteristic after complete relaxation when the energy density of the pump light is increased. Our results are consistent with those of previous studies on the ultrafast kinetics of other vanadium oxide films, which show the response of films to the pumping light of different energy densities [36,37,38].

## 4. Conclusions

V_2_O_5_ films were prepared on sapphire, magnesia, and silica substrates. The optical properties were studied by a UV–VIS–NIR spectrophotometer and spectroscopic ellipsometer. The morphological characteristics were checked by AFM, and their main components were tested by XPS and EDS. The phase transition temperature of the sample measured using a four-point probe method was 260 °C. The ultrafast photo-induced phase transition of V_2_O_5_ films was investigated by the femtosecond laser pump–probe method. It was found that V_2_O_5_/Al_2_O_3_ film has better phase transition characteristics compared to the V_2_O_5_ films of the other two substrates because it can complete the transformation in a shorter time of 160 fs. The recovery time of the V_2_O_5_/SiO_2_ film after phase transition was 100 ps, which was shorter than that of the V_2_O_5_ films on the other two substrates, and it showed excellent recovery characteristics. V_2_O_5_/Al_2_O_3_ film induced by a higher energy density laser will have a faster phase transition speed and a greater degree of phase transition. This is due to the formation of more free electrons in the V_2_O_5_ structure. Our results will help strengthen the general understanding of photo-induced phase transitions in V_2_O_5_ films on different substrates and facilitate the application of all-optical ultrafast switches or memory devices.

## Figures and Tables

**Table 1:** Phase transition characteristics of V_2_O_5_ thin films obtained by ultrafast pump–probe method.

V_2_O_5_ Samples	Sample 1	Sample 2	Sample 3
Substrates	Al_2_O_3_	MgO	SiO_2_
Energy intensity (mJ/cm^2^)	0.0622	0.0622	0.0622
Phase transition time t_1_ (fs)	160	200	200
Initial transmittance T_1_ (%)	79	85	80
Phase transition transmittance T_2_ (%)	65	47	60
T_1_ − T_2_ (%)	14	38	20

**Table 2:** Fit parameters *A*, *B,* and *C* for Equation (1) and recovery characteristics.

V_2_O_5_ Samples	Sample 1	Sample 2	Sample 3
Substrates	Al_2_O_3_	MgO	SiO_2_
Energy intensity (mJ/cm^2^)	0.0622	0.0622	0.0622
*A*	243.60	−15.39	−4.60
*B*	0.0028	−0.3979	−0.6398
*C*	−169.20	86.75	80.93
Recovery time t_2_ (ps)	200	150	100
Recovery transmittance T_3_ (%)	78	84	80

## Data Availability

Not applicable.
